# Highly Sensitive Trace Gas Detection Based on In-Plane Single-Quartz-Enhanced Dual Spectroscopy

**DOI:** 10.3390/s22031035

**Published:** 2022-01-28

**Authors:** Tiantian Liang, Shunda Qiao, Ziting Lang, Yufei Ma

**Affiliations:** National Key Laboratory of Science and Technology on Tunable Laser, Harbin Institute of Technology, Harbin 150001, China; 21S021041@stu.hit.edu.cn (T.L.); 20B921022@stu.hit.edu.cn (S.Q.); 20B921021@stu.hit.edu.cn (Z.L.)

**Keywords:** quartz tuning fork (QTF), quartz-enhanced photoacoustic spectroscopy (QEPAS), light-induced thermal elastic spectroscopy (LITES), trace gas detection

## Abstract

For this invited manuscript, an in-plane single-quartz-enhanced dual spectroscopy (IP-SQEDS)-based trace gas sensor was demonstrated for the first time. A single quartz tuning fork (QTF) was employed to combine in-plane quartz-enhanced photoacoustic spectroscopy (IP-QEPAS) with light-induced thermoelastic spectroscopy (LITES) techniques. Water vapor (H_2_O) was chosen as the target gas. Compared to traditional QEPAS, IP-SQEDS not only allowed for simple structures, but also obtained nearly three times signal amplitude enhancement.

## 1. Introduction

Trace gas detection has numerous significant applications in various fields, such as biomedical analyses [1,2], combustion diagnosis [3,4,5], atmospheric environmental monitoring [6,7,8,9], as well as petroleum exploration [10,11]. Gas sensing technology based on laser absorption spectroscopy (LAS) has a number of advantages, including high sensitivity, rapid response time, high selectivity, et al. [12]. One of the most commonly used techniques of LAS for gas sensing is photoacoustic spectroscopy (PAS). In PAS, gas samples absorb the modulated or pulsed laser and then release heat outward by non-radiative transition, leading to local thermal expansion and generating acoustic waves. The intensity of external acoustic waves is related to the concentration of gas and can be investigated using a sensitive microphone. Nevertheless, the low resonance frequency and low Q factor (<100) of microphones cause poor signal-to-noise ratios (SNRs) for the sensor system. In addition, the microphone-based PAS also possesses the larger size of photoacoustic cells.

**Figure 1:**
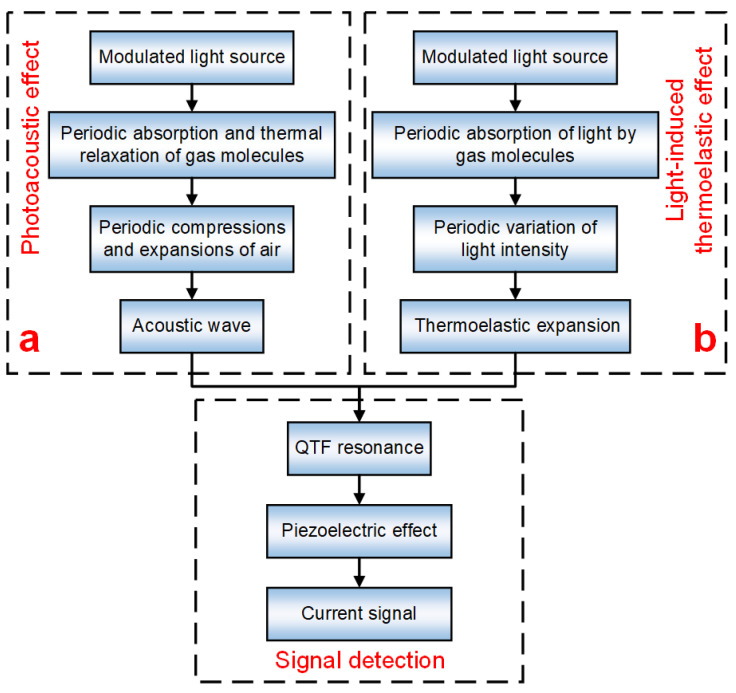
Functional block diagram: (**a**) QEPAS; (**b**) LITES.

An effective improvement method for PAS techniques was firstly put forward in 2002 by taking the place of microphones with a piezoelectric quartz tuning fork (QTF) [13], in which the QTF serves as an acoustic wave detector, whose principle is shown in Figure 1a. This technique is called quartz-enhanced photoacoustic spectroscopy (QEPAS). QEPAS offers a new method for trace gas detection, which provides the merits of high Q-factor detection of QTF (~100,000 in a vacuum and ~10,000 under standard atmosphere pressure), strong anti-noise ability, compact volume and inexpensive price. Hence, various QEPAS-based sensors have been developed in recent years for meeting detection requirements of different trace gases [14,15,16,17,18,19].

In-plane quartz-enhanced photoacoustic spectroscopy (IP-QEPAS) was proposed by Ma et al. in 2020 [20]. Unlike traditional QEPAS, IP-QEPAS varies the incident position of laser beams. The direction of the laser beam is altered from perpendicular to the QTF plane to parallel to it. Therefore, it improves the effective interaction path length between target gas molecules and excitation sources. Compared to traditional QEPAS, IP-QEPAS had a signal enhancement of more than 40 times when a custom QTF was used [20].

In 2018, a new technique of light-induced thermoelastic spectroscopy (LITES) was firstly reported [21]. In LITES, a QTF is employed to detect the absorption variation of light intensity [22,23,24,25,26]. For LITES, whose principle is shown in Figure 1b, when the quartz at the surface of the QTF is irradiated by an intensity modulated laser, the quartz crystal absorbs the laser and converts it into photothermal energy. As a result of this characteristic of light-thermo-elastic conversion, the QTF will undergo elastic deformation, which results in the mechanical vibration of the QTF and the generation of piezoelectric signals [27,28]. Moreover, the oscillation amplitude and the obtained piezoelectric signal will reach a maximum when the intensity-modulated frequency of light is equal to the resonant frequency of one of the flexural modes of the QTF. Compared to QEPAS, LITES can be a non-contact measurement technique [29,30,31]. Therefore, it can be used in some harsh environments, such as combustion fields. Gold and silver are generally applied as a coating on QTFs to collect the electrical charges generated by them; they surely provide excellent conductivity as well as strong reflectivity to light. In the traditional LITES technique, the laser is usually first illuminated on the QTF coating, and then transmitted to the quartz, which results in the low variation of the amount of light absorption by the quartz and further leads to the QTF’s low sensitivity [32,33]. It is worth noting that the standard, commercially available QTF is usually coated with silver only on the surface, and it is not coated in the middle of the two prongs [15,21].

In this invited article, an in-plane single-quartz-enhanced dual spectroscopy (IP-SQEDS) gas detection technique is reported for the first time, in which the laser beam is made parallel to the QTF plane and finally incident to the uncoated quartz at the bottom of the QTF, so that QEPAS and LITES signals are simultaneously excited in a single QTF. An in-plane configuration was adopted to take full advantage of SQEDS. Water vapor (H_2_O) was adopted as the analyte to evaluate the IP-SQEDS-based sensor performance.

## 2. Experimental Section

**Figure 2:**
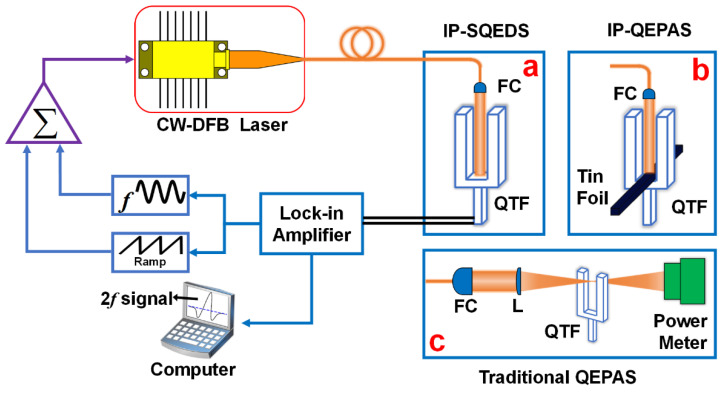
Schematic diagram of experimental design: (**a**) IP-SQEDS; (**b**) IP-QEPAS; (**c**) traditional QEPAS.

The experimental setup of the IP-SQEDS sensor is displayed in Figure 2. A standard, commercially available QTF with a length of 6 mm, a width of 0.6 mm, a thickness of 0.36 mm, and a gap between the two prongs of 0.3 mm was adopted. The laser beam output from the pigtail of a CW-DFB diode laser was collimated by a fiber collimator (FC). Subsequently, as shown in Figure 2a, in order to improve the effective interaction path length between target gas molecules in the QEPAS technique and excitation sources and enhanced light absorption of QTFs in the LITES technique, the laser beam was passed through the space between the two prongs of the QTF and eventually made incident to the uncoated quartz at the bottom of the QTF. The generated acoustic wave and the periodically absorbed laser energy produced QEPAS and LITES signals, respectively. In order to confirm that the total signal was produced by processes of both QEPAS and LITES, the experimental setup containing only IP-QEPAS was designed by replacing Figure 2a with Figure 2b. A tin foil was placed near the bottom of the QTF and used to only eliminate the LITES signal. In contrast, in the traditional QEPAS sensor, as shown in Figure 2c, the laser beam was collimated by a fiber collimator (FC), and then focused and passed through the optimal location of 0.7 mm below the top of the two prongs of the QTF [18] to obtain the strongest response.

**Figure 3:**
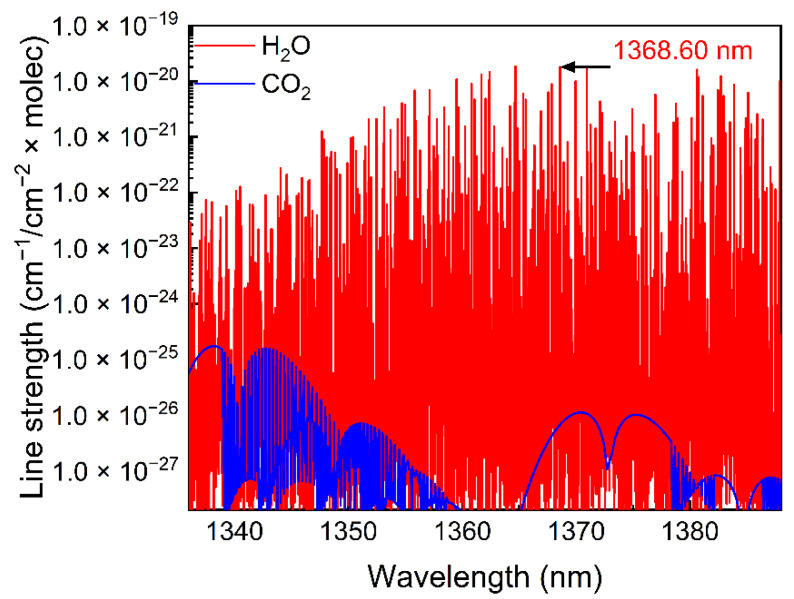
Simulations of line strength based on the HITRAN 2016 database.

H_2_O naturally exists in the atmosphere and is non-toxic and harmless [34]. Therefore, at room temperature, H_2_O with a volume concentration of 1.73% naturally existing in the air was adopted as the analyte to evaluate the performance of this IP-SQEDS-based sensor. According to the HITRAN-2016 database [35], the line strength of H_2_O and CO_2_ located between 1335 nm and 1385 nm was simulated and is shown in Figure 3. It can be seen that the absorption line of H_2_O situated at 1368.60 nm (7306.74 cm^−1^) has a strong line strength while avoiding the interference from CO_2_.

**Figure 4:**
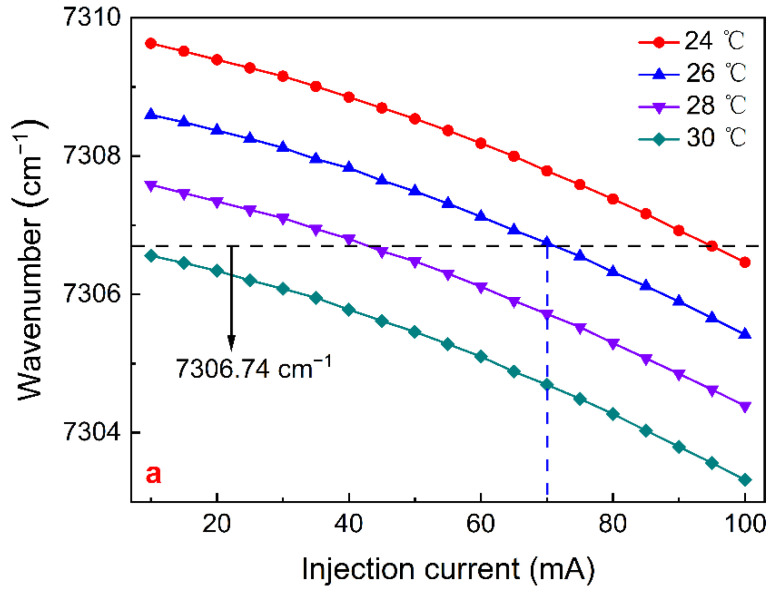

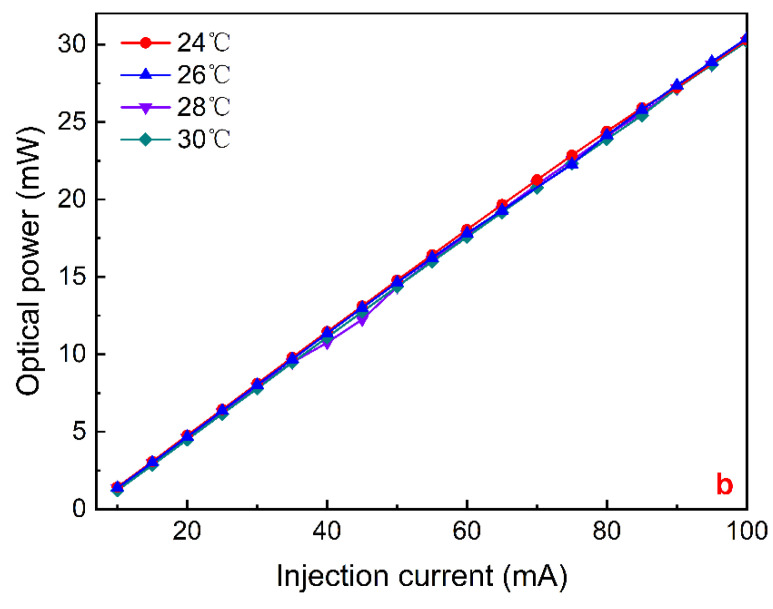
Output characteristics of the 1368.60 nm CW-DFB fiber-coupled diode laser: (**a**) output wavenumber as a function of injection current at four different temperatures; (**b**) optical output power as a function of injection current at four different temperatures.

A continuous wave (CW), distributed feedback (DFB), fiber-coupled diode laser emitting at 1368.60 nm was applied as the excitation source. The wavenumber and output power as a function of the injection current are shown in Figure 4a,b, respectively. The working temperature and injection current of this CW-DFB laser was set to 26 °C and 70 mA, respectively, to ensure that its emission wavelength matched the absorption wavenumber at 7306.74 cm^−1^ of H_2_O. The emitted optical power was ∼19.72 mW.

Wavelength modulation spectroscopy (WMS) was performed to modulate the output wavelength of the diode laser, and second-harmonic (2*f*) detection technology was utilized to demodulate the produced signals from the QTF. The above functions were implemented by employing a Zurich lock-in amplifier. On the one hand, the lock-in amplifier generated a saw-tooth signal and a sine signal. The signal obtained by superposition of the two signals was transmitted onto the laser controller to modulate the output wavelength of the diode laser. On the other hand, the lock-in amplifier extracted the target signal with a particular frequency. These methods effectively maximized the amplitude of the 2*f* signal and minimized the low-frequency and irrelevant noise of the experimental system.

## 3. Results and Discussion

**Figure 5:**
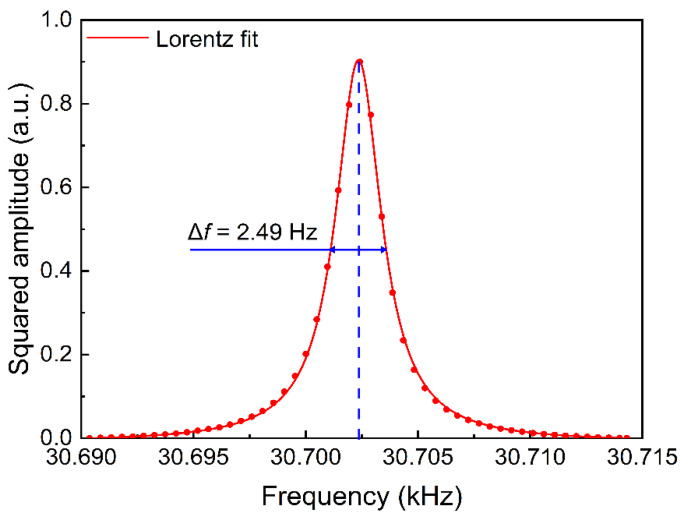
The frequency-response curve of the QTF. These data have been normalized and fitted by a Lorentz function.

Firstly, the properties of the QTF were determined. The resonance curve of the QTF was measured and is shown in Figure 5. The inherent resonant frequency *f*_0_ of the QTF was measured as 30,702.4 Hz by using an optical excitation approach, while full width Δ*f* was 2.49 Hz at half-maximum. Based on the formula, *Q* = *f*_0_/Δ*f*, the quality factor (*Q*-factor) was determined as 12,330. The frequency of the sine signal provided by the lock-in amplifier was set as *f*_0_/2 = 15,351.2 Hz to obtain the maximum 2*f* signal amplitude.

**Figure 6:**
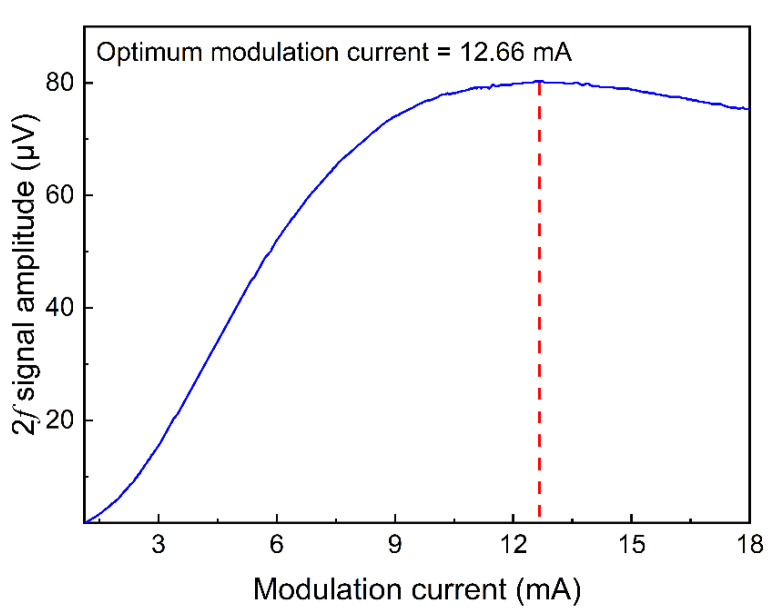
IP-SQEDS signal amplitude as a function of modulation current depth (H_2_O concentration was 1.73%).

Figure 6 reflects the relationship between laser wavelength modulation current and IP-SQEDS signal amplitude. As the modulation current depth increased, the IP-SQEDS signal amplitude rose at first, but then tended to flatten, and finally declined slowly. The maximum signal was obtained when the modulation current depth was 12.66 mA. As a result, this value was considered as the optimal modulation current and continued to be applied in the subsequent research.

**Figure 7:**
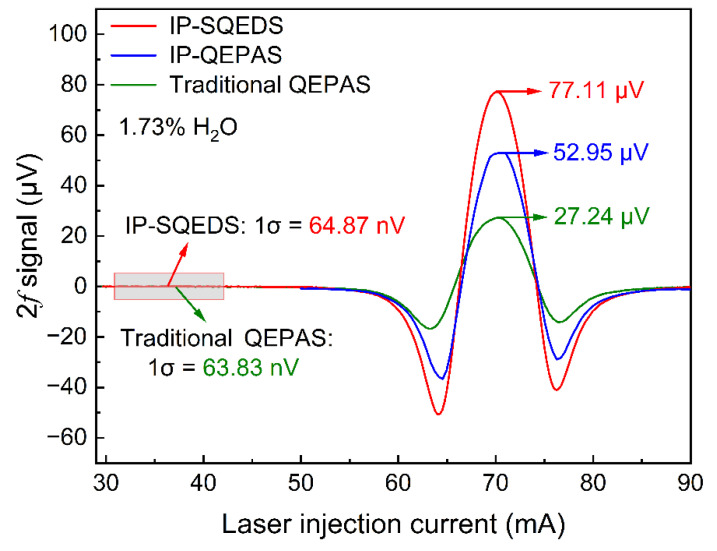
2*f* signals of IP-SQEDS, IP-QEPAS and traditional QEPAS.

The 2*f* signals for IP-SQEDS, IP-QEPAS and traditional QEPAS are shown in Figure 7; the experimental configurations of Figure 2a–c were respectively adopted and the time constant of the lock-in amplifier was set as 20 ms. The maximum 2*f* signal amplitude of IP-SQEDS and traditional QEPAS were measured as 77.11 and 27.24 μV, respectively. The noise was determined when the laser wavelength was far away from the H_2_O absorption line. The 1σ noise levels of traditional QEPAS and IP-SQEDS were almost the same and measured as 63.83 and 64.87 nV, and their SNRs were 426.76 and 1188.69, respectively.

In order to verify that the signal level of 77.11 μV contained both IP-QEPAS and LITES, a slim tin foil was utilized as the obstacle and placed near the bottom of the QTF to maintain the IP-QEPAS signal and eliminate the LITES signal. Experimental results are shown in Figure 7. The maximum IP-QEPAS 2*f* signal amplitude was measured as 52.95 μV. It is proven that the signal amplitude of 77.11 μV was generated from IP-QEPAS and LITES together. Compared to traditional QEPAS, IP-SQEDS had a signal enhancement of nearly three times. It can be calculated that the minimum detection limit (MDL) of the H_2_O IP-SQEDS sensor was ~14.55 ppm.

## 4. Conclusions

In summary, this paper proposed a method of IP-SQEDS for the first time, which combines IP-QEPAS and LITES by using a single QTF. H_2_O with a volume concentration of 1.73% was selected as the target gas and the absorption line located at 1368.60 nm (7306.74 cm^−1^) was chosen. A standard, commercially available QTF with a resonant frequency of 30,702.4 Hz and a Q factor of 12,330 was adopted in the experiments. The maximum 2*f* signal amplitude of IP-SQEDS was measured as 77.11 μV, while that of the traditional QEPAS was 27.24 μV. In addition, a slim tin foil was chosen as an obstacle and placed near the bottom of the QTF for eliminating only the LITES signal. It is proven that the signal of 77.11 μV contained both IP-QEPAS and LITES. Compared to traditional QEPAS, this IP-SQEDS sensor possessed a simply constructed and compact structure and resulted in a signal enhancement of nearly three times. In the future, on the premise of maintaining the U-shaped structure of QTFs, further improvements in the performance of IP-SQEDS can be expected by optimizing the structure of the QTF, such as the length of the two prongs and the size or form of uncoated quartz between them. 

## Data Availability

The data presented in this study are available on request from the corresponding author.

## Abbreviations

LAS	Laser Absorption Spectroscopy
PAS	Photoacoustic Spectroscopy
QTF	Quartz Tuning Fork
QEPAS	Quartz-Enhanced Photoacoustic Spectroscopy
IP-QEPAS	In-plane Quartz-Enhanced Photoacoustic Spectroscopy
LITES	Light-induced Thermoelastic Spectroscopy
IP-SQEDS	In-plane Single-Quartz-Enhanced Dual Spectroscopy
